# Biological physics by high-speed atomic force microscopy

**DOI:** 10.1098/rsta.2019.0604

**Published:** 2020-10-26

**Authors:** Ignacio Casuso, Lorena Redondo-Morata, Felix Rico

**Affiliations:** 1 Aix-Marseile University, Inserm, CNRS, LAI, 163 Av. de Luminy, 13009 Marseille, France; 2 Center for Infection and Immunity of Lille, INSERM U1019, CNRS UMR 8204, 59000 Lille, France

**Keywords:** high-speed force spectroscopy, single molecules, biophysics, membranes, proteins, cells

## Abstract

While many fields have contributed to biological physics, nanotechnology offers a new scale of observation. High-speed atomic force microscopy (HS-AFM) provides nanometre structural information and dynamics with subsecond resolution of biological systems. Moreover, HS-AFM allows us to measure piconewton forces within microseconds giving access to unexplored, fast biophysical processes. Thus, HS-AFM provides a tool to nourish biological physics through the observation of emergent physical phenomena in biological systems. In this review, we present an overview of the contribution of HS-AFM, both in imaging and force spectroscopy modes, to the field of biological physics. We focus on examples in which HS-AFM observations on membrane remodelling, molecular motors or the unfolding of proteins have stimulated the development of novel theories or the emergence of new concepts. We finally provide expected applications and developments of HS-AFM that we believe will continue contributing to our understanding of nature, by serving to the dialogue between biology and physics.

This article is part of a discussion meeting issue ‘Dynamic *in situ* microscopy relating structure and function’.

## 1. Introduction

The dialogue between physics and biology has existed since the first experiments by Helmholtz on the mechanics of the eye and the discoveries by Galvani and Volta on electrical stimuli of frog muscles [1,2]. The dialogue continued with the speculative analysis of Schrodinger and the discovery of the structure of DNA, evolving into a field in itself through the application of optical traps to biomolecules [3–7]. In the era of quantitative biology, the application of new tools to obtain physical understanding of biology seems necessary. Moreover, as the most fundamental questions in physics reach a stalled point, turning the eyes into biological systems in search of emergent physical phenomena seems inevitable [8]. Biological physics involves the study of biological systems from which physical concepts and principles emerge and stand independently of the original system [9–11]. Biological processes are subjected to thermal fluctuations at the nanoscale and provide a gigantic playground to explore emergence of physical phenomena. On the one hand, single-molecule optical microscopy techniques are particularly well suited to study the dynamics of molecules with excellent time resolution but are limited to labelled protein regions [12]. On the other hand, high-resolution techniques like X-ray crystallography and cryo-electron microscopy had an important impact in the field of structural molecular biology but they provide static images based on ensemble averaging [13]. The apparition of nanotools has boosted the field of biological physics at the nanoscale. The first experiments on single biomolecules opened the door towards physical understanding of biomolecules, the stepping mechanisms of molecular motors or the binding strength of receptor/ligand bonds [14–22]. Furthermore, nanotechnologies allowed direct verification of fundamental fluctuation–dissipation theorems on single biomolecules in a paradigmatic example of biological physics [5,23].

The atomic force microscope has since its invention in 1986 [24] quickly been positioned among the single-molecule, high-resolution structural analysis techniques. Atomic force microscopy (AFM) is now in its thirties and an established methodology among the biophysical community as a stand-alone, high-resolution imaging technique and force transducer [25]. AFM provides pN force sensitivity and nanometre resolution and thus allows mechanical measurements and high-resolution imaging in liquid, at ambient temperature and pressure, being optimal for biological applications. While the contribution of AFM as an imaging tool to biological physics has been relatively limited, as a force tool AFM has been very useful. The major drawback of AFM is the relatively low temporal resolution, requiring minutes to obtain a single image and limiting force measurements to approximately 0.1 milliseconds. The development of ultrashort cantilevers with microsecond time resolution and fast electronics and piezoelectric scanners allowed 1000-fold faster imaging rates to be reached, which led to the emergence of what now is named high-speed AFM (HS-AFM). The development and application of HS-AFM imaging is introducing a new dimension to structural biology: time [26,27]. HS-AFM allows the visualization of conformational changes of proteins in real time. HS-AFM imaging has been used to study a number of biological systems, such as the activity of proton pumps, the dynamic interaction of proteins with DNA, ligand-induced conformational changes in ion channels, and the diffusion and assembly of membrane and scaffold proteins [28–33]. Moreover, the flexibility of biomolecules has also been reported on antibodies showing the capacity of these essential molecules to adapt their shape for better binding to the target [34]. Moreover, ultrashort HS-AFM cantilevers enable high-speed force spectroscopy (HS-FS) measurements with µs time resolution to explore fast protein dynamics, challenge theoretical predictions and allow direct comparison with molecular dynamics (MD) simulations.

In this review, we tried to focus on contributions of HS-AFM on biological physics, i.e. on physical phenomena that occur in biological systems, at the nanoscale. While the emergence of HS-AFM is relatively young, an exhaustive review of all published works would be enormous. Thus, the present review is not meant to be comprehensive and we already apologize for the missing works that could not fit here. The chosen HS-AFM works we report have been driven, unavoidably, by personal interest but also because they represent examples in which physics is central, leaving the biological system itself as secondary. Since some of the physical concepts may not be familiar, we have tried to provide a short introduction to the topic. First, we briefly describe the main developments allowing HS-AFM. We then focus on applications of HS-AFM to biological systems and processes in which physics plays a major role: dynamic organization of membranes, molecular machines, dynamic (un)folding of proteins and single-molecule mechanics. The review includes both applications of HS-AFM imaging and HS-FS.

## 2. HS-AFM

The first time that the concept of high-speed AFM was mentioned—to our knowledge—was in 1991 by Barrett & Quate [35], who provided a fair attempt with the technology available at that time. The groups of Hansma and Ando would challenge the practical speed limitations [36,37]. In 2010, Ando's group filmed individual myosin molecules walking on an actin filament [38], operating their HS-AFM instrument at a speed about 1000 times faster than conventional AFM systems. Besides the visual impact and scientific insight of those movies, these experiments illustrated that HS-AFM was able to obtain concomitantly structural and dynamic data of biomolecules, providing insights inaccessible by any other method.

### (a) High-speed imaging and force spectroscopy

**Figure 1.**
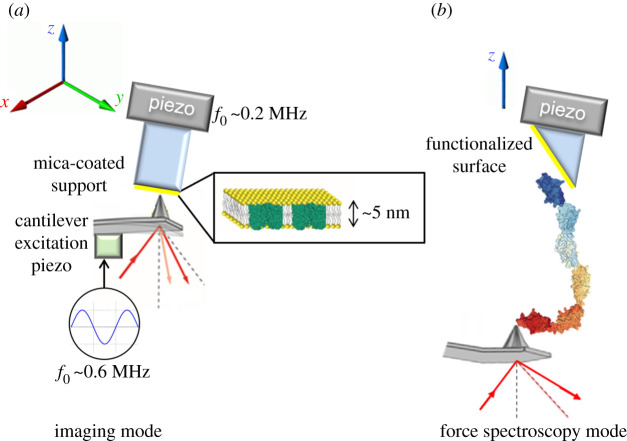
Schematics of HS-AFM systems for imaging (*a*) and force spectroscopy (*b*). For high-speed AFM imaging, the cantilever is excited through a small piezo element near its resonance frequency (approx. 0.6 MHz in aqueous solution). For flat surfaces such as biomembranes (schematized in the inset with outer membrane protein F pdb code: 3POQ) the sample is scanned in the horizontal plane (*xy*) and the cantilever deflection is monitored by a photodetector, which collects the reflection of a laser beam focused at the back of the AFM cantilever. Topographical images are obtained from the continuous correction of the z movement to keep the amplitude of oscillation constant using a feedback controller. In the case of force spectroscopy mode, the sample approaches to and retracts from the sample in the vertical direction and the force between the tip and the sample is determined from the cantilever deflection. The sample may be tilted to further reduce the effective viscous drag coefficient of the cantilever near the surface. Functionalized surfaces and cantilever tips are often used to probe the mechanics of single molecules, such as multidomain proteins (the I-band fragment I65–I70 from titin, pdb code: 3B43, is shown). (Online version in colour.)

AFM modes that have an intermittent tip-sample contact are commonly used for the study of biological samples to minimize damage and mechanical perturbation. The most often intermittent contact-mode used is acoustic-modulation (tapping) mode, in which the AFM cantilever is excited at its resonance frequency. The resulting oscillating tip is intermittently contacting—tapping—the surface, which consequently damps the oscillation amplitude. The surface topography is reconstructed by monitoring the amplitude of the oscillating cantilever. While scanning in the *x*-*y* plane, the amplitude of the cantilever oscillation is kept constant thanks to a proportional–integral–derivative feedback controller that controls the *z* position. HS-AFM is nowadays almost exclusively operated in tapping-mode to minimize the force or amount of energy transferred at the tip-sample contact. The maximum rates of HS-AFM range between 5 and 20 frames per second (fps) [39]. While different approaches have been introduced to allow fast scanning rates [40,41], the miniaturization of the moving components of the AFM (cantilever and scanner) and faster electronics are the basis of the technical developments which allow reaction responses of microseconds and consequently to increase the imaging rate [42]. In brief, the most important elements that allow AFM to be operated at a video-rate are (figure 1):

(1) *Ultrasmall cantilevers*. Modern microfabrication techniques allow the cantilever dimensions to be reduced to 7 µm long, 2 µm wide and 90 nm thick (considerably smaller than conventional cantilevers, 40–200 µm long, 20–30 µm wide and 400–800 nm thick). This is probably the most critical point for HS-AFM imaging: the reduction of the cantilever mass allows high resonance frequencies of approximately 500 kHz in liquid and short response times (approx. 1 µs) to be achieved. Long amorphous carbon deposits are grown on the cantilever, which serve as a tip once etched.
(2) *Fast actuators*. The *z*-piezo scanner is driven by high-frequency signals during high-speed imaging, which generate mechanical vibrations. To minimize the vibrations, a dummy piezo is placed in the opposite direction and actuated simultaneously in order to counterbalance the impulsive force. Furthermore, z-scanners are individually calibrated, and the resonance frequencies filtered out. The *x*- and *y*-piezo actuators—larger than the *z*-piezo—keep their centres of mass stationary using flexure stages and by attaching a balance weight to the counter side. They are also embedded in a silicon elastomer to passively damp vibrations.
(3) *Fast amplitude detectors*. The cantilever oscillation amplitude is measured and output by a Fourier method at every cycle of the oscillation.
(4) *Adaptive/dynamic feedback*. During fast imaging, the tip tends to ‘detach’ completely from the surface at the downhill regions, an effect known as ‘parachuting’. To address this, a dynamic controller was developed, consisting of a feedback controller that increases automatically the gain when downhill regions are scanned.

HS-AFM has also been adapted to perform HS-FS. When AFM is operated in force spectroscopy mode, the tip approaches and retracts perpendicularly to the surface at controlled force and velocity. This allows detection of specific interactions using functionalized tips and probing of mechanical properties of single molecules, membranes and other living cells. The miniaturization of the cantilever in HS-AFM results in a very low viscous damping, which is a prerequisite for a µs-response time. In fact, the components and their miniaturization necessary to perform HS-FS are equivalent to those of imaging. Another technical particularity to improve the performance of HS-FS is to tilt the surface 45°, minimizing the effective viscous drag of the cantilever. With this configuration, it is possible to reach pulling velocities up to approximately 30 mm s^−1^ [43,44]. Such recordings required analogue-to-digital converter with high temporal resolution (tens of megasamples/s). Other technical modifications have been explored allowing the use of new methodologies with the HS-AFM set-up for improved mechanical measurements. Smaller *z*-piezo actuators give access to high frequencies (up to 120 kHz) in order to probe the microrheology of soft samples at high rates [45]. The use of torsional harmonic cantilevers also allows force measurements of receptor/ligand bonds with µs-time resolution [46]. Micromachining of ultrashort cantilevers also remarkably improve force sensitivity while maintaining µs response time [47].

Particularly in the case of operation on cells, AFM systems are usually coupled to optical microscopes. This helps us to position the AFM cantilever over the cell and to visualize major changes. In order to visualize the cells while maintaining the performance of HS-AFM, a prism was used on top of the *z*-piezo, where the sample stage is mounted. This allowed transmitted illumination of the sample [45,48]. In that case, a ‘sample-scanning’ set-up was used, in which the sample moves with the piezoelectric components while the cantilever is fixed—this configuration is mechanically stable. Nowadays, there are already commercial ‘tip-scanning’ HS-AFM set-ups. In this case, the tip moves relative to the sample and therefore an inverted optical microscope can be easily coupled, allowing advanced approaches such as total internal reflection fluorescence microscopy [49].

Still, HS-AFM remains a surface technique, so the molecules of interest need to be adsorbed or immobilized on a substrate. This, in turn, may restrict the dynamics and natural interaction between biomolecules. Even so, molecules diffusing faster than the frame rate cannot be imaged by means of HS-AFM. Thus, for each system to be studied by HS-AFM, the conditions of sample, medium and substrate need to be optimized.

Combination of FS and imaging modes leads to approaches such as force mapping in which force curves are acquired at different locations across the sample surface to obtain multiparametric maps revealing, for example, elasticity or adhesion [50]. While the application of HS-AFM for force spectroscopy-based mapping is still not mature, some recent works point in this direction [33,51–53].

## 3. Biological physics explored by HS-AFM

### (a) Dynamic organization of membranes

The cell membrane is the fundamental support matrix and ultimate energetic barrier of the cell. In biological membranes lipids mainly organize in lamellar phases, where acyl chains of two different lipid leaflets align oppositely to form a hydrophobic core and the polar headgroups remain exposed to the water interface. Since the discovery of lamellar phases in cell membranes [54], many attempts have been made–and are still being made–to explain the lateral heterogeneity of membranes through relevant physical–chemical features. The two most outstanding hypotheses in the field are the fluid-mosaic model by Singer and Nicolson in 1973, depicting cell membranes as two-dimensional liquids where all lipid and protein molecules diffuse easily [55], and the lipid-rafts hypothesis proposed in 1997 by Simons and Ikonen [56], which depicted functional lipid patches—rafts—in the two-dimensional fluid matrix. Nowadays, it is well known that there are a variety of nanostructures in the membrane of heterogeneous sizes and functions, and the methods that allow us to observe these nanodomains *in vivo* are only starting to emerge [57]. HS-AFM represents now a major tool to study the dynamic organization of biological membranes.

#### (i) Phase transition of lipid membranes

Comparable to cell membranes, biomimetic membranes, typically studied as supported lipid bilayers (SLBs) or vesicles in suspension, adopt different phases: solid (gel phase) and fluid (liquid-ordered and liquid-disordered phases). The occurrence of these phases depends on the lipid nature, temperature and other environmental conditions. First-order transitions of lipid membranes are well known; at low temperatures lipids are arranged on a triangular lattice, known as solid phase (or gel phase or *L_β_*). By contrast, at high temperatures lipids are in the liquid phase (or fluid phase or *L_α_*), reflecting the order–disorder transition of their hydrocarbon chains. Some lipids present a pretransition before the main first-order transition which gives rise to an intermediate order in the lipid bilayers referred to as ripple phase (P*_β_’*). Ripple phases are smectic phases characterized by structural periodic corrugations. AFM imaging revealed the structure of ripple phases in hydrated conditions at the nanometre scale [58]. The observation of a phase transition process is characterized by a melting point temperature of the ripple phase to fluid phase, nucleation point and growth directionality for the formation of the ripple phase.

HS-AFM coupled to a temperature-controlled system reported directly ripple to fluid phase transitions (reversibly) in real time and at high resolution [59]. When the fluid bilayer was cooling down, ripples appeared as concentric rings progressing from the edge region of the lipid patch towards the centre upon cooling. This ring-pattern formation was maybe a consequence of a heterogeneous thermalization along the nanometric patch and cooling progressed from the edges that exposed the largest surface to the cooling bulk. It could also be a consequence that the bending and edge formation of the lipids at the patch border represented favourable nucleation spots for ripple phase reformation, or both. In fact, throughout cycles of heating/cooling, the ripple phase pattern changed every time, suggesting that the thermal history of the bilayer was erased in the fluid phase. Based on the van't Hoff expression, the representation of area fraction of each phase as a function of the temperature provides estimations about the transition enthalpy and the cooperativity of the lipid molecules during the process. The cooperativity of each process also accounts for the order of the transition, more cooperative processes relate to first-order transitions, where the latent heat is required to change the phase, while in second-order transitions heat is also necessary for changing the temperature. Ripple phase melting occurs at locations where ripple pattern interfaces (disorders) are detected. These disordered areas probably comprise a slightly higher amount of liquid-like lipid molecules and hence present ideal locations for initiation of melting or nucleation of the meta-stable ripple phase. Furthermore, the local transition of the bilayer into fluid phase, i.e. a complete mixing of the lipids at high lateral dynamics, leads to a ‘loss of memory’ of the bilayer. The correlation of nanoscale thermotropic transitions with micro and mesoscopic thermodynamic descriptors, as the enthalpy of the process, helps to further understand the system. A lipid membrane containing small domains does not necessarily correspond to a phase coexistence regime, but it may constitute a one phase regime with micro- or nanoscale heterogeneities. In this sense, HS-AFM observations may help fill the gap between single-molecule diffusion studies to the collective migration of lipid domains or patches. This could have implications for more complex cellular systems where lipid nanodomains form and dissociate reversibly.

#### (ii) Fractality

The cell membrane is an intrinsically complex and dynamic bidimensional fluid densely crowded with proteins on which ordered patterns do not dominate. Individual membrane proteins participate at more than one function. Therefore, by combination, the number of functions of the membrane proteins is higher than the number of membrane proteins. Fractal geometry is a useful mathematical tool to understand the interaction of multiparticle systems and characterize systems with several characteristic lengths or levels of construction. Fractal geometry is defined by the fractal dimension (*D*). For membrane proteins, the more fragmented the spatial distribution of the proteins, the higher *D*. Fractal geometry has, for example, been used in biology to describe plants or cardiovascular vessels. It has been shown that higher values of fractality reduce the energy required for the distribution of the resources [60–62]. At the molecular scale, the role of fractality is less well understood. At the cell membrane, the assessment of fractality has been ‘blind’ and indirect, calculated from models and observations of diffusing biomolecules tagged with fluorescence markers [63,64]. The basic idea is that the geometric partitioning of the membrane (dangling ends, bottlenecks and backends) generates restricted areas of diffusion and interaction and inter-connectivities between the membrane proteins that have dramatic effects on the biochemical kinetics of the cell membrane. Mathematically, on fractal arrangements, the number of sites that a random walker molecule visits in *N* motion steps is proportional to the fractal dimension (so, the number of visited sites ∼*N^D^*) [65]. Therefore, in the areas of the membrane of partitioning of higher fractality, the protein diffusion searches ‘more carefully’ the space and minimizes the chances of missing a target in the close vicinity.

The HS-AFM concomitant visualization of diffusion and the surroundings is an ideal tool to study the fractality of the plasma membrane and the correlation of protein–protein interactions with the organization of the membrane [66]. HS-AFM videos of the spatio-temporal distribution of the bacterial channel outer membrane porin F (OmpF) on membranes [67] showed that the trimers of OmpF arrange in an intricate geometry with a high fractal dimension *D *≈ 1.73 ± 0.01, a value of *D* characteristic of particles performing a random walk and sticking together the moment they touch. This process of fractal formation known as diffusion limited aggregation (DLA) [68,69] provides a wide variety of local environments that promote, for example, translocon complexes [70] or that ensures that the fluxes of matter that cross the OmpF are evenly distributed across the bacterial plasma membrane and arrive consuming minimal energy to the entire cell. For comparison, a simulation of the OmpF trimers arranged in a two-dimensional array had a fractal dimension of *D *≈ 1.05 + 0.02, so the OmpF arrangements expose about twice as much molecular surface as molecules in a regular array. Besides DLA, another physical process that can produce fractal-like membrane partitioning is percolation. In particular, when membrane domains fuse to a single continuous one (percolation threshold), fractality appears at short distances, but the distribution is homogeneous at lager length scales, two different temporal diffusion regimes appear at this moment across the membrane [71]. HS-AFM may lead to the observation of other fractality processes by allowing dynamic visualization of all the components of a biological system at the nanoscale.

#### (iii) Glassy mosaic model

As described above, fractality is a possible approach to quantify the complexity of a system. Two disciplines have evolved side by side in our comprehension of natural complexity: biology and condensed matter physics, yet, on the topic of glasses they are far apart. In physics, the glass transition and glass rheology has been for years one of the ultimate and most blurry and undefined frontiers and the literature in this respect is vast [72]. On the contrary, biology mentions of glasses are sporadic at best. Probably, scale is the most relevant factor that explains such discrepancy. Glass rheology experiments track particles in the micrometre to millimetre scale, where particles are easy to visualize. By contrast, protein tracking experiments using fluorescence microscopy take place at the nanoscale where tracking is challenging. In addition, the spot size of the fluorescence tag used to track biomolecules, approximately 200 nm, is too large to distinguish the collective motions of neighbouring molecules. Because glasses originate in collective dynamics that emerge when particles cluster in a short time creating a long-lasting non-equilibrium, glass dynamics is difficult to identify using fluorescence particle tracking [64]. Another factor that may have kept glasses out of the radar of biology is that the trajectory of a particle in a glass looks (at experimentally available times) just like the trajectory of the biomolecules interacting with their surroundings, leading to what is known as anomalous diffusion.

**Figure 2.**
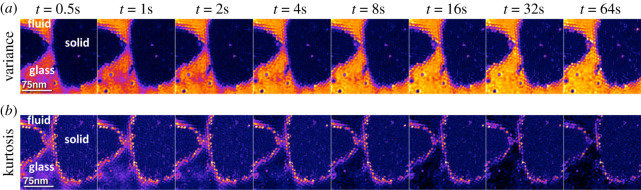
Glassy states in biological membranes. Areas of non-Brownian dynamics of lysenin toxin on supported lipid bilayers identified by HS-AFM topographic movies. The different time-lags show variation of the (*a*) variance (V) of the distribution of height changes (false colour scale: 0 < *V* < 2 nm) and (*b*) kurtosis (*K*) (non-Gaussianity) of the distribution of the height changes (false colour scale: 2.5 < *K* < 5.0). Adapted from [73]. (Online version in colour.)

HS-AFM offers a unique possibility to assess glassy states in biological membranes as it enables video imaging of the totality of molecules in the ensemble. Using HS-AFM [73], it was shown that glassy states appear in biological membranes on assemblies of lysenin, a pore forming toxin from the coelomic fluid of the earthworm *Eisenia fetida*. Lysenin binds and readily forms clusters in the lipidic membrane domains of the targeted cells [73–75]. HS-AFM data showed that the glassy state interconnects membrane regions of solid and fluid character. In the glassy regions, cages of neighbours obstruct the diffusion with a characteristic time, a few seconds, which regulates the flow of particles across the glassy zones. Diffusion was non-Gaussian at lag-times smaller than the characteristic time and Gaussian at longer lag-times. Such glassy areas were observed by HS-AFM to surround the solid 2D crystal and, thus, to define the exchange rate with the free Gaussian diffusion regions (figure 2).

We can ask ourselves what evolutionary advantage an organism could obtain from the use of glassy states. We can think that the organisms could create glassy states to stop and reactivate their molecular ensembles without any requirement for large structural transitions, as is the case if phase transitions occur, or as a molecular clock that ticks at the pace of ageing of collective phenomena. As a matter of fact, some molecular trajectories detected in living cells, described using the formalism of continuous-time random walk [76], which considers a continuous distribution of waiting times between trajectory steps, could be due to the diffusion of the molecules in a glassy state [77]. Future experiments may allow us to observe other properties of glassy systems, such as ageing, that may lead to the description of the membrane as a *glassy mosaic model*.

#### (iv) Protein self-assembly during fission membrane remodelling

The potential of HS-AFM technology transformed our understanding of protein self-assembly in membranes. During membrane remodelling, there are—generally speaking—two sorts of events, fusion and fission, which give rise to topological changes. The topological invariant is described by the Euler number, which is decreased during fusion, when two vesicles merged, or increased during fission, when two vesicles divide. Membrane remodelling events are then classified based on the topological changes and the location during the remodelling process [78]. Thus, external changes are led by proteins located on the external leaflet of the membrane budding neck, and internal changes are those driven by proteins which assemble in its lumen.

**Figure 3.**
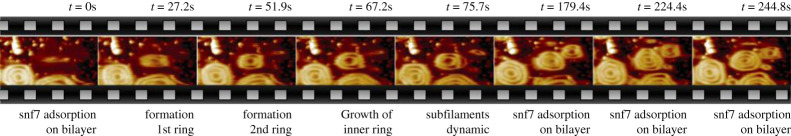
HS-AFM movie frames showing how Sn7, the major polymerizing component of ESCRT-III, assembles as a flat spiral disk on flat lipid membranes. Snf7 spirals can function as spiral springs. Using HS-AFM, the polymerization energy and the rigidity of Snf7 filaments were estimated, showing that they were deformed while growing in a confined area. The elastic expansion of compressed Snf7 spirals could stretch the lipids they are bound to, generating an area difference between the membrane leaflets and thus curvature. This spring-like activity underlies the driving force by which ESCRT-III could mediate membrane remodelling. For insights, see [79]. (Online version in colour.)

Concerning internal fission in membrane remodelling, a successful HS-AFM study was focused on the endosomal sorting complex required for transport-III (ESCRT-III) [79]. This complex-based machinery is highly evolutionarily conserved, and it is required for lipid membrane remodelling in many cellular processes, from abscission to viral budding and multi-vesicular body biogenesis. Snf7, the major component of ESCRT-III, polymerizes as a spiral transiently in the membrane (figure 3), inducing curvature which eventually will contribute to bud and constrict the membrane. The model proposed in [79] for deformation and budding induction relies on the elastic relaxation of the ESCRT-III polymer. HS-AFM observations showed that Snf7 flexible filaments have a preferred curvature radius, hence growing as a flat spiral onto the lipid membrane they accumulate elastic stress. The external filaments of the spirals-disks are underbent: the more it polymerizes, the higher the energy it accumulates. By measuring the polymerization energy and the rigidity of Snf7 filaments, HS-AFM videos showed that the elastic expansion of compressed Snf7 spirals could stretch the lipids they are bound to, generating an area difference between the membrane leaflets (buckling) and, thus, inducing curvature. This spring-like activity of ESCRT-III is, to our knowledge, new to the field of membrane remodelling. HS-AFM also provided high-resolution films of the enzymatic disassembly of ESCRT-III by the ATPase Vps4 [80]. In the presence of a pool of soluble Snf7, ESCRT-III assemblies shrank under the action of Vps4, liberating free space on the membrane where new ESCRT-III assemblies were growing simultaneously. This results in turnover and high lateral mobility of ESCRT-III assemblies on membranes. Dynamic turnover provides an explanation for how ESCRT-III filaments gradually adapt their shape during membrane constriction, which has broad implications in diverse cellular processes, differing in size, shape and duration—such as plasma membrane repair, cytokinesis or viral budding.

External fission has been extensively described for the dynamin machinery, which assembles as a helix in the external part of the budding neck. Dynamin is a GTPase motor and its constriction is essential for cell events such as endocytosis and organelle division. However, the mechanism of constriction and twist by the dynamin helix has been thoroughly debated. The complexity arises from the fact that the dynamin polymer has both contractile and torsional abilities, involving changes of contiguous dimers at the molecular level but also at the whole polymer level—changes not accessible by standard structural biology tools. HS-AFM allowed the visualization of the constriction of single dynamin-coated membrane tubules [81,82], showing the distance between the helix turns and between dimers along the polymer. These distances were shown to vary over time, as helical turns were observed to transiently pair and dissociate. This hampers the propagation of constriction along the length of long helices. Eventually, local fission occurs only where constriction is the strongest. At fission sites, these cycles of association and dissociation were correlated with relative displacement of the turns and constriction. HS-AFM findings support a model in which conformational changes at the dimer level drive relative sliding of helical turns, and constriction by torsion.

The application of HS-AFM on biological membranes contributed to our understanding of biological function through physical descriptions of the molecular processes and, importantly, allowed the observation of emergent physical phenomena.

### (b) Molecular machines

The last examples reveal the importance of chemical energy-driven processes in biological function. The proteins that run most of these processes are often known as molecular motors. Molecular motors are the scientific leitmotif that drove Toshio Ando at the University of Kanazawa to invest a decade of efforts in the development of HS-AFM. Molecular motors are biomolecules capable of generating directed motion by taking energy from the statistical fluctuations that surrounds them, thanks to an asymmetric energy landscape with respect to the spatial direction of displacement. In addition, the activity cycle of molecular motors includes one or several irreversible gating steps (that convert energy of low entropy into higher entropy energy), forcing the motor to move forwards. Even if the ruling principles of the physics of molecular motors are known, each motor has evolved to develop its own strategies of implementation, which adapt better to a specific function. Many details of their functioning are controversial and different experiments have resulted in different conclusions. Many of these discrepancies originate from the lack of simultaneous measurement of the conformational and chemical changes of molecular motors.

One of the motors whose mechanism of functioning is still unclear is myosin V. Myosin V puzzles because the experimental data suggests that the gating step in its mechanism takes place in the opposite sense to what would be expected from its directional motion: it takes place simultaneously to the attachment of the motor to the filament, a favourable event that does not require additional free energy contributions. Why myosin V supplies even more energy at this step is unclear. Other molecular transport motors, like kinesin, use the free energy supplement of ATP hydrolysis to detach the motor from the molecular track. The predominant hypothesis is that myosin V stores the energy supplied by the ATP hydrolysis in the form of mechanical energy creating intermolecular stress. The activity of myosin V was filmed at the molecular scale using the HS-AFM by Ando *et al*. [83]. This was the first-ever detailed assessment of the time-lapsed sequence of structural configurations of myosin V, out of reach of other techniques previously used to characterize molecular motors (fluorescence microscopy, laser tweezers, X-rays or electron microscopy). The HS-AFM data provided new insights into the debated myosin V mechanism of action: it showed that the creation of intermolecular stress in the myosin V structure could be induced by the simple attachment to the actin filament, and that no extra free energy from the ATP was required. HS-AFM data suggests that the free energy supplement could be exclusively required to break the bond formed to the actin filament, as in the case of the kinesin motor. At the present time, the precise mechanism of myosin V remains unclear.

ATP synthases are rotary catalytic molecular machines ubiquitous in organisms from plants to amoeba to humans that produce ATP from ADP. During its rotation the ATP synthase modifies the shape of its enzymatic pockets, and there are three structural-enzymatic steps related to the different rotation angles (the binding of an ADP and a phosphate, the attachment of the phosphate, and the release of ATP). The free energy required for the synthesis of ATP from ADP is provided by the flow of protons that moves across the molecule (the proton-motive force). Taking advantage of the HS-AFM fast imaging, it was possible to conclude that the F1 domain of the ATP-synthase is capable of performing its structural transitions independently of the F0 domain. The HS-AFM movies show isolated F1 domains undergoing the structural transitions of its activity cycle [84]. It had previously been hypothesized that the rotation of F0 controlled the enzymatic activity of F1. HS-AFM showed that F1 can structurally cycle independently of F0. The HS-AFM video imaging also enabled the first-ever AFM imaging of ATP-synthases on bacterial membranes diffusing on close proximity to the proton-pump machinery (the bacteriorhodopsin proteins) and supramolecular organizing in short-lived assemblies of two molecules (dimer) [85]. The oligomerization of the ATP-synthase is believed to help its function as by a mutual compensation of the torque forces the rotors exert on the membrane [86]. Moreover, in this work, the fast frame rate and the nanometre resolution of HS-AFM provided a method to determine membrane-mediated interaction of integral membrane proteins, a method not accessible to other tracking techniques.

Another interesting molecular motor is the helicase domain of DNA-modifying enzymes, such as restriction enzymes. During DNA translocation, this motor puts in contact two distal DNA sites. HS-AFM observations revealed two mechanisms of communication of the two DNA sites: passive diffusion of the protein over the DNA—which depends, in turn, on the DNA persistence length—and specific protein binding [87]. The capacity of HS-AFM to visualize dynamic conformational changes in proteins is currently shifting our perspective of structural biology by introducing the dimension of time.

### (c) Dynamic (un)folding of proteins

A functional protein, such as myosin V described above, often requires the folding of the polypeptide chain into the secondary and tertiary structure. Understanding of this folding process is a challenge in biology, but also from a physical point of view. Already in 1968, Cyrus Levinthal proposed a paradox that suggested that the time required for a protein to fold into its native state from a random exploration of its configurational space should be exceptionally long [88]. However, it is known that proteins can fold within seconds or less. While different mechanisms have been proposed, the process by which proteins find their native folded state is still largely unknown and represents a tempting challenge for physicists. Indeed, a large number of works have tried to decipher the folding or unfolding process of proteins using different experimental and theoretical tools.

An important conceptual advance in our understanding of protein folding was the introduction of the concept of funnel-shaped, rough energy landscape by Frauenfelder *et al*. [89]. Their early experiments used photolyzing over nine decades in timescale to observe the rebinding of CO to myoglobin [89,90]. The authors were able to observe the relaxation and the conformational changes undergone by the protein upon rebinding of CO in a process that resembled quakes. This led to the concept of a multi-tier, rough energy landscape in which proteins jump from substate to substate thanks to thermal motion. The concept took advantage of advances in the field of spin glasses, revealing again the importance of the dialogue between physics and biology [91]. The funnel shape ensures that the protein is directed towards substates of lower and lower energy avoiding frustration [11]. This idea allows us to somehow reconcile the mechanism of protein folding in a similar way, with the protein diffusing down the energy landscape in search of the native state. As we will see below, the concept of energy landscape will allow us to interpret protein (un)folding processes probed by HS-AFM.

#### (i) Intrinsically disordered proteins

**Figure 4.**
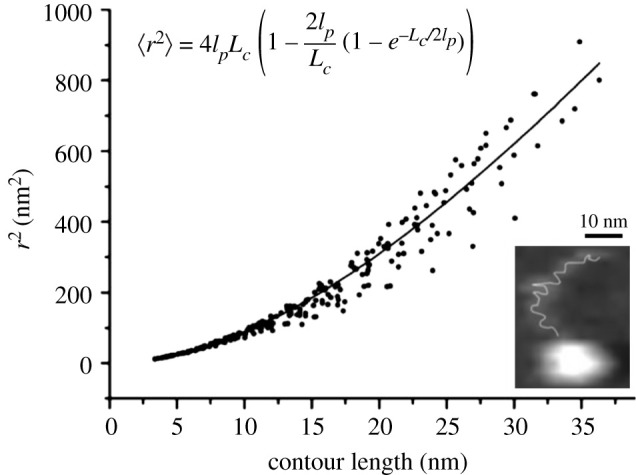
Intrinsically disordered FACT proteins visualized by HS-AFM imaging. The mean squared point-to-point distance (*r*) of tail-like structures measured from movie frames (insert) as a function of the contour length (*L*_C_) allows determination of the macroscopic persistence length (*l*_p_) using the appropriate chain polymer model (equation). The insert shows a movie frame revealing a FACT protein with the contour length of the tail-like region schematically depicted. Adapted from [94].

While most known proteins present a folded state, intrinsically disordered proteins (IDPs) represent a large family of proteins whose structure is not stable and most of the polypeptide chain is disordered. In addition, structured proteins often contain disordered regions, which appear as blurred electron density maps in X-ray or cryoEM data. IDPs have even been defined as ‘protein clouds’ invoking their dynamic nature and lack of secondary or tertiary structure, but as an ensemble of structural conformations not directly accessible in conventional high-resolution structural approaches [92]. Study of these particular proteins is still a challenge. HS-AFM is likely the only technique capable of visualizing the dynamic structure of IDPs [93]. An important contribution towards a visualization of unfolded proteins was reported on IDPs [93–95]. In the work by Miyagi *et al.*, the authors studied facilitates chromatin transcription protein (FACT), a protein with predicted large ID regions that facilitates RNA polymerase II transcription and chromatin remodelling [94]. HS-AFM imaging at a remarkable 5–17 frames per second of immobilized FACT revealed a stable lumpy structure, flanked by long tail-like structures that underwent rapid fluctuations (figure 4). Analysis of the contour length of the fluctuating regions and using deletion mutants allowed them to identify the tail structures as the two major ID regions. Moreover, analysis of the root mean squared point-to-point distance of the tail-like structures as a function of the contour length provided a measure of the elasticity of these ID regions by using statistical polymer chain analysis to obtain the persistence length (*l*_p_). Interestingly, the reported *l*_p _∼ 10 nm was much larger than the value usually obtained in force-extension curves of unfolded proteins (approx. 0.4 nm), suggesting higher stiffness [96–98]. Making IDPs stiffer (larger *l*_p_) would indeed lower the probability of reaching compact, folded structures by limiting the number of accessible states. This is likely accomplished through electrostatic repulsion between charged residues, abundant in IDPs [99]. IDPs seem to have evolved to avoid a funnel energy landscape and remain within a tier of states with similar energy, still avoiding misfolding and, perhaps, taking advantage of frustration [100].

#### (ii) Forced protein unfolding at µs time scales

**Figure 5:**
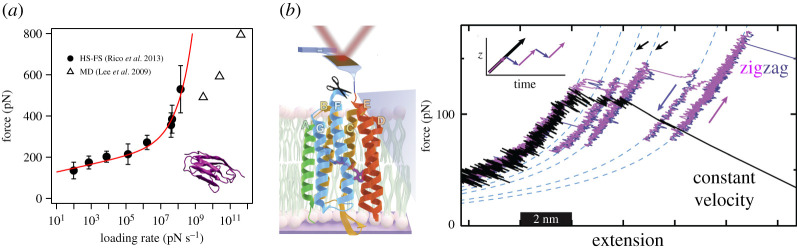
High-speed force spectroscopy. (*a*) DFS of titin I91 domain unfolding from HS-FS experiments [43] and MD simulations (open triangles, [103]). The inset shows the crystal structure (pdb code: 1TIT). The red solid line is the best fit to the BSK model with parameters *k*^0^ = 7 × 10^−6^ 1 s^−1^, *x*_b _= 0.45 nm and *ΔG*^‡ ^= 25 *k*_B_*T*. (*b*) Bacteriorhodopsin unfolding from zigzag experiments using modified ultrashort cantilevers (left). Force-extension curve revealing different intermediate states being visited during both extension (purple) and release (black) trajectories (adapted from [104]). (Online version in colour.)

Another approach to studying folding and unfolding of individual proteins is force spectroscopy (FS) [21,96,101]. The adaptation of HS-AFM now allows FS experiments at high velocities with µs time resolution [102]. Unlike AFM imaging that tries not to perturb the proteins, FS of protein unfolding consists in grabbing a protein with the probe, pulling from it at constant velocity and measuring the force required to unfold it. Early experiments by the group of Hermann Gaub revealed that certain protein domains, such as the immunoglobulin-like (I) domains of titin formed by *β* sheets, unfolded in an abrupt manner in a reversible processes that was described with just two states: the native, folded state and the unfolded state [21]. Being a thermally activated process, unfolding forces depended on the pulling rate, actually on the rate of force increase or loading rate (figure 5). The Bell-Evans model allows us to model this process and characterize the energy barrier in terms of a distance to the transition state (*x*_b_) and an intrinsic unfolding rate (*k*^0^) from the fit to the spectrum of unfolding force versus loading rates [105]. A large number of experiments have shown that, while the intrinsic unfolding rate ranges several orders of magnitude, the distance to the transition state lies within a narrow range of values between approximately 0.1 nm to approximately 2 nm [96]. The interpretation of this distance from a structural point of view is still a matter of debate and reaching a microscopic understanding will certainly require a combination of MD simulations. Indeed, MD simulations of forced unfolding provide an atomic description of the process. However, the computational cost of MD simulations did not allow pulling at the same velocity applied in experiments, therefore not allowing direct comparison of the unfolding forces [106]. In an effort to bridge this gap, HS-AFM was adapted to allow HS-FS [43]. The first HS-FS experiments pulled on the I91 domain of titin at velocities up to approximately 4 mm s^−1^, more than 100 times faster than conventional AFM, reaching the lower range of velocities probed by all atom MD simulations. The unfolding forces measured over five decades in pulling velocity revealed a logarithmic response with an upturn at the highest velocities and predicted an energy landscape with *x*_b _∼ 0.89 nm and *k*^0^ ∼ 2×10^−10^ s^−1^ (0.45 nm and 7 × 10^−6^ s^−1^ if using the loading rate, figure 5). This distance was remarkably larger than earlier results (approx. 0.25 nm) but closer to the extension at which titin loses its tertiary structure (1.1–1.4 nm) reported by MD simulations [103,106]. HS-FS was also applied to unfold spectrin repeats, α-helical domains. In contrast to titin, HS-FS revealed a distance of approximately 0.5 nm, much lower than the distance at which tertiary structure breaks (approx. 5 nm) as revealed from MD simulations [107]. It is important to note that other theoretical models may lead to different values of *x*_b_ and *k*^0^ and that several combinations of these two parameters may lead to good fits [108]. Thus, while theoretical models allow us to obtain a phenomenological picture of protein unfolding, only the combination with MD simulations will provide the necessary hints to a microscopic interpretation [109]. As we will address below, only a dialogue between experiments, theory and simulations will reconcile microscopic and phenomenological interpretations.

An advantage of single-molecule techniques is the capacity for observing transient events and exploring intermediate states that may be averaged down in bulk measurements. Intermediate states have been observed using FS, including on titin and spectrin [110,111]. The application of HS-FS represented a step forward in our understanding of these intermediate states. In the first study reporting intermediates of titin I91, the authors combined AFM with MD simulations, which allowed them to interpret a pronounced hump before complete unfolding during extension as the disruption of a β-strand, that extended approximately 0.7 nm. Later studies revealed that the force at which this hump appeared was rate-independent and acted as a force buffering mechanism to prevent titin domains from complete unfolding [112]. HS-FS confirmed this rate-independent behaviour up to a certain velocity, upon which the force followed the usual logarithmic dependence [43]. These results were interpreted as two different dynamic regimes: a near-equilibrium regime in which folding and unfolding competed, and a thermally activated regime above a certain velocity, at which refolding became negligible due to the high force reached. A similar mechanism was found for spectrin repeats in which the dynamic equilibrium between unwinding and rewinding of α-helices resulted in a force plateau in unfolding force-distance curves [107]. This observation was supported by MD simulations which revealed unwinding/winding events at the slowest velocities. These works showed the dynamic nature of small secondary structures such as β-strands and α-helices, which constantly assemble and disassemble due to thermal motion in dynamic equilibrium, equilibrium broken by mechanical force.

Membrane proteins are particular because they live within the lipid bilayer of the membrane and mechanical unfolding also involves extraction from the lipid environment. In an early work, Oesterhelt *et al.* used AFM to image and mechanically extract and unfold bacteriorhodopsin (bR), an α-helix rich protein that works as a proton pump in purple membranes [113]. The results revealed that the unfolding pathways of bR correlated well with the orientation and organization of the protein, with two pairs of helices (GF and ED) unfolding pairwise and another pair (BC) occasionally unfolding sequentially. Interestingly, the authors already predicted that ‘better instruments should allow an even more detailed interpretation of the unfolding pathways'. Indeed, recent work by the Perkins group used ultrashort HS-AFM cantilevers modified using focused ion beam (FIB) to unfold bR [114]. FIB modification of ultrashort cantilevers significantly enhances the force sensitivity while keeping µs response time [47]. This allowed the authors to reveal more complex bR unfolding pathways. While the original work on bR suggested 3 or 4 intermediate states, Yu *et al.* identified as many as 14 intermediate states only in the unfolding of the ED helix pair. Moreover, the relatively slow pulling velocity applied (300 nm s^−1^) and the improved temporal and force resolution allowed observation of quasi-equilibrium fluctuations between states separated only by one α-helical turn and with dwell times down to 8 µs (potentially 3 µs). Furthermore, by retracting the cantilever at predefined steps, equilibrium experiments at constant force allowed the back and forth transitions of a single alpha-helical turn within approximately 15 µs to be visualized. These equilibrium trajectories allow them to reconstruct the energy landscape for the unfolding of one α-helix turn, resulting in an unusually high energy barrier of approximately 4.7 k_B_T. Notably, this work resolved long-standing discrepancies between conventional AFM experiments and MD simulations [115], again corroborating that the complementarity of experiments and simulations is optimally achieved if both techniques probe the system at similar time scales. In an intelligent approach, Jacobson *et al.* applied zigzag extension curves to the same bR system to allow repetitive sampling of the various intermediate states, significantly enhancing the statistics and revealing previously hidden states [104] (figure 5). It would be interesting to see if the number and location of the visited states remains constant or rather varies by changing loading rate as simulations predict [115]. As pointed by the authors [114], HS-FS studies seem to challenge the notion that most SMFS experiments of protein unfolding occur far from equilibrium, while refolding has been observed in titin, spectrin and bR unfolding. Temporal resolution, thus, matters and provides a better understanding of the complex process of protein folding.

### (d) Single-molecule mechanics at the shortest timescales: bridging experiments, theory and simulations

As seen in the previous section, experiments, theory and MD simulations have walked together since the beginning of SMFS. The first FS experiments determining the rupture forces of (strept)avidin/biotin (SA/b) bonds were rapidly followed by theoretical developments and MD simulations that allowed us to interpret the reported rupture forces [17,105,116,117]. On the one hand, theoretical models provided a phenomenological understanding based on the concept of energy landscape. As described above for protein unfolding, unbinding was also modelled as a barrier crossing process from the bound to the unbound state, in which the barrier height was lowered by the applied force. This allowed the dependence of the unbinding/unfolding force as a function of loading rate to be predicted. The Bell-Evans model established a logarithmic dependence of force versus loading rate that allowed the determination of the energy landscape parameters *x*_b_ and *k*^0^ [105,118]. Finally, MD simulations provided a description of the atomic details. MD simulations can be seen as a nanoscopic attainment of Laplace demon in which the equations of motion of all the atoms of the system are numerically solved at each time step (usually of 2 fs) [103]. The series of time steps result nowadays in µs-long trajectories with accurate knowledge of the relevant events during unbinding or unfolding processes. Due to the high computational cost, in the case of SA/b, the original MD simulations were carried out at pulling velocities orders of magnitude higher than in experiments, thus covering very short time scales (ps-ns). This posed an interpretation problem because the resulting simulation forces were much higher than the experimental ones. The seminal work by Evans and Ritchie already tried ‘to bridge the enormous gap in time scales between MD simulations and laboratory experiments’ by proposing different dynamic regimes for experiments and simulations. Indeed, different works proposed, in addition to the thermally activated regime of experiments, a drift or deterministic ultrafast regime at the much faster velocities of MD simulations [105,116,119]. In the ultrafast pulling regime, the force would increase, not logarithmically, but as the square root of the loading rate. These works suggested that the forces extracted from experiments and from simulations were not directly comparable, as the theoretical assumptions and approximations depended on the pulling rate. As shown above, HS-FS using ultrashort cantilevers allowed *de facto* bridging of the gap between experiments and simulations [43,103]. The reported curved shape of the dynamic force spectrum (figure 5) suggested that the deterministic regime for titin I91 was reached at a critical force of approximately 350 pN, or a critical loading rate of 10^7^ pN s^−1^. The critical loading rate is defined by the parameters of the energy landscape, as $\dot{F_{c}}=DF_{c}/x_{b}^{2}$, where $F_{\text{c}}=2\Delta G^{\text{‡}}/x_{\text{b}}\;$ is the critical force at which the barrier disappears, *D*, the diffusion constant and Δ*G*^‡^, the free energy barrier height. This implies that the intrinsic properties of the system define the transition from thermally activated to ultrafast regime. Indeed, HS-FS experiments on spectrin repeats reported a critical loading rate of approximately 10^10^ pN s^−1^, three orders of magnitude faster than that for titin I91 domains. Soon after this work, a theoretical development by Bullerjahn, Sturm and Kroy proposed an analytical model (BSK) covering both thermally activated and deterministic regimes [120]. Remarkably, the work considered all possible experimental settings in terms of pulling regimes, spring constants and initial conditions, and improved our understanding of the dynamic force spectra obtained from experiments and simulations. The disappearance of the barrier at a critical force represents, however, a conceptual problem (see electronic supplementary material of ref. [120]). Indeed, when the barrier disappears, it is difficult to define the actual point of barrier crossing, and thus the transition from bound to unbound or folded to unfolded. Moreover, at this point the high-barrier approximation by Kramers is no longer valid. Other theoretical developments allow description of the curvature in the dynamic force spectrum based only on the shape of the energy landscape or the presence of multiple barriers, however, they fail at the critical force [121–126]. The recent Cossio–Hummer–Szabo (CHS) theoretical model is worth noting for its capacity to describe a wide dynamic range covering experiments and simulations using a single barrier [127]. The authors introduced the concept of kinetic ductility to describe the mechanical response of single molecules. Ductility, as opposed to brittleness, allows the energy landscape to gradually stretch upon applied force before barrier crossing. That is, on a perfectly brittle molecule *x*_b_ remains constant upon force application, while on a perfectly ductile molecule, *x*_b_ will shrink indefinitely with applied force. This allowed the description of the full HS-FS spectrum of I91 unfolding as a thermally activated process in which the Kramers approximation of high barrier holds for a much wider dynamic range, without reaching the deterministic regime. The actual shape and brittleness of the energy landscape is nonetheless only accessible using additional data, like bulk determined *k*^0^, or provided a wide range of loading rates is available, only possible combining HS-FS and MD simulations. Furthermore, the kinetic ductility model requires a remarkably high diffusion coefficient (or preexponential factor). As shown in the force versus loading rate spectrum (figure 5), the last value at the highest experimental velocity deviates from the expected trend. This deviation was interpreted in the same work by Cossio *et al*., suggesting that at the highest pulling rates of HS-FS, the limited response time of the cantilever may overestimate the measured forces [127]. Future experiments will verify this prediction. It is, however, curious that the use of cantilevers with the shortest response time raised the question of its very influence in FS experiments. This suggests that pushing the technological limits makes science advance at a faster pace [128].

Both works on titin and spectrin using HS-FS allowed reconsideration of other theoretical models. In the case of titin, HS-FS revealed that the forces of β-sheet unfolding (hump) were constant for a range of pulling velocities, for then increasing logarithmically at higher rates. In the case of spectrin, a similar behaviour was observed for the continuous uncoiling process of the α-helices. Given the small dimensions of these two structures, this dynamic behaviour was interpreted in terms of an unfolding/refolding quasi-equilibrium regime at which off and on rates competed. The theoretical model by Friddle, Noy and de Yoreo described this process but considering a virtual state due to the convolution of outer barrier of the energy landscape with the inner barrier of partial unfolding [129]. Again, MD simulations were in agreement with the experimental results. This reinforces the idea that the combination of HS-FS and MD simulations allows a back and forth dialogue between the two techniques framed by theoretical models.

HS-FS has also been used to measure the binding strength of receptor/ligand interactions. In the recent work on streptavidin/biotin (SA/b) unbinding, the rupture forces were determined using HS-FS and MD simulations over 11 decades of loading rate [44]. HS-FS probed the forces required to break the streptavidin/biotin bond at velocities up to 30 mm s^−1^, reaching a loading rate of approximately 10^9 ^pN s^−1^, which overlapped with the slowest velocity simulations. The tetrameric form of streptavidin bound to biotin was used and biotin was pulled through a worm-like chain potential linking the biotin to a virtual spring with the same stiffness as in the experiments. Thus, simulations mimicked the exact experimental conditions. Again, at overlapping rates, rupture forces showed excellent agreement. Interestingly, the very same simulations on the monomeric form of streptavidin showed poorer agreement, reflecting the advantage of combining experiments and simulations at the very same time scales. The full dynamic force spectrum was not entirely described by a single barrier model but required multiple barriers [44]. Brownian dynamics simulations of a double barrier predicted a critical loading rate of approximately 10^11^ pN s^−1^, only accessible to MD simulations. This extremely fast critical value is reasonable given the small biotin molecule rapidly diffusing across a short distance before unbinding. While the proposed energy landscape was able to describe the unbinding force spectrum of SA/b over 11 decades of loading rate, it predicted an off rate at zero force of ∼1 s^−1^ incompatible with the off rate obtained on bulk experiments (∼1 days^−1^). This advocated for another mechanism that would obstruct and slow down the exit of biotin from the SA pocket, after the second barrier was crossed. Indeed, both HS-FS experiments and MD simulations suggested outer barriers due to side-chain interactions and transient induced fits of SA, all depending on the loading rate. Thus, streptavidin may have evolved to slow down the fast diffusion of biotin outside the binding pocket, favouring rebinding and bringing to the complex its unusually long lifetime. Importantly, the outcomes suggested revisiting old and well-established concepts like the lock-and-key model describing receptor/ligand bonds in terms of rate-dependent induced fits of the protein dynamically wrapping around the moving ligand.

The physical description of the mechanics of cells is inevitably linked to polymer physics, given the polymeric network of the cell cytoskeleton. While single-molecule mechanics is difficult to probe on cells, HS-FS has also been applied to living cells in a series of experiments probing the microrheology of the cytoskeleton at high frequencies [45]. In this work, Rigato *et al.* recovered the well-known viscoelastic response of living cells described by soft glassy rheology at low frequencies (less than 0.1–1 kHz) and extended the viscoelastic range up to 100 kHz. In this high-frequency regime, the response was expected to be dominated by the dynamics of the individual cytoskeletal filaments. Indeed, the reported power-law exponents were consistent with predictions from semiflexible filament theories. In this case, the atomic description of the system using MD simulations is still far given the large size of the system, but coarse-grained models may allow us to better understand this dynamic behaviour [130]. We hope that HS-FS will provide a framework to better understand the inherently dynamic nature of single-molecule mechanics by bringing together experiments, theory and simulations.

## 4. Conclusion and future perspectives

We have reviewed some examples in which physical phenomena emerge from biological systems and whose description and characterization were less clear before the emergence of HS-AFM. HS-AFM has filmed biomolecules at subsecond framerates, perhaps sufficient for capturing the details of a number of processes, like diffusive crowdedness or macromolecular cooperativity, but too slow for capturing many other biomolecular processes. Such is the case, that even the iconic HS-AFM movie of the walking myosin V required slowing down the motion of the legs by introducing obstacles [83]. Thus, faster HS-AFM approaches, likely revising the probe technology, will be required to capture the dynamics of other biomolecular processes, such as the gating of ion channels and the diffusion of small proteins [131]. As reviewed here and elsewhere, the energy landscape of proteins and receptor/ligand bonds is rough, with a hierarchy of substates that are transiently and dynamically visited, substates that can now be directly explored on single molecules using HS-FS at µs-temporal resolution. Future experiments would allow us to determine if this dynamic equilibrium is coloured by dynamic disorder and heterogeneity [132,133]. It may also allow the detection of fast, transient folded states in IDPs or monitor the complete process of protein folding, where short-lived frustration events may occur, and glassy dynamics may dominate [134]. Moreover, future combination of other nanotools with HS-AFM imaging may allow the observation of forced unfolding in real time [26]. The certainly upcoming development of high-speed force mapping will allow us to address dynamic modulation of the mechanics in biological systems, such as proteins, membranes and cells [51]. Proteins and membranes seem to share similarities at different length and time scales and physical concepts such as rough energy landscape and frustration provide a phenomenological understanding of these systems. As computational power increases, MD simulations at experimental, µs time scales will become the norm. Combination of conceptual frameworks with experiments and MD simulations will allow us to reach microscopic description of biological physics phenomena. The dialogue between physics and biology is expected to last and HS-AFM will provide a new tool to moderate it.

## Data accessibility

This article does not contain any additional data.
